# Epidemiology of Pediatric Musculoskeletal Trauma Patients Admitted to a Trauma Center in Northern India: A Prospective Cohort Study

**DOI:** 10.7759/cureus.43327

**Published:** 2023-08-11

**Authors:** Vikas Verma, Mayank Mahendra, Devarshi Rastogi, Abhishek Agarwal, Syed Faisal Afaque, Prajwal M C

**Affiliations:** 1 Department of Pediatric Orthopedic Surgery, King George's Medical University (KGMU), Lucknow, IND; 2 Department of Orthopedics, King George's Medical University (KGMU), Lucknow, IND; 3 Department of Sports Medicine, King George's Medical University (KGMU), Lucknow, IND; 4 Department of Orthopedic Surgery, King George's Medical University (KGMU), Lucknow, IND

**Keywords:** epidemiology, injury, fracture, musculoskeletal, pediatric trauma

## Abstract

Background

Pediatric injuries are the leading cause of death and disability worldwide and place a considerable burden on nations with limited resources. A careful investigation of the epidemiology of pediatric musculoskeletal trauma can provide insight into its causation and the demography of the affected children and help us devise preventive strategies to reduce the burden of pediatric musculoskeletal trauma.

Methodology

Musculoskeletal trauma patients up to the age of 18 years were included in this prospective cohort study. Information about age, sex, time since the injury to presentation to a trauma center, mode of injury, the site where the injury was suffered, and the exact injury were recorded. Age was further recorded as 0 to 3 years, >3 to 6 years, >6 to 12 years, and >12 to 18 years. A subgroup analysis of the mode of injury was done using age group and sex.

Results

A total of 201 patients were enrolled in the study. The age (mean ± standard deviation [SD]) of the enrolled patients was 12.48 ± 4.71 years. Of the 201 patients enrolled, 146 (72.63%) were males. The mean time since the injury to the reception in the emergency department of the King George's Medical University trauma center (a tertiary care center) was 19.13 ± 33.86 hours. The common mechanisms of injury observed were road traffic accidents (RTAs, 55.22%), falls from height (29.35%), and falls at ground level. There was a significant difference in the mode of injury in the age groups (*P *= 0.0297) and among males and females (*P *= 0.0034). Injuries to the lower limbs were most common in all age groups.

Conclusions

Our study presents the baseline epidemiological data on pediatric musculoskeletal injuries distributed by age groups, gender, mode of injury, site of injury, and region-wise distribution of injuries. The data may be used by policymakers in planning a pediatric trauma care system in India.

## Introduction

Trauma has been defined as the injury sustained as a result of blunt/penetrating harm due to external forces like road traffic accidents (RTAs), poisoning, falls, drowning, etc. [1,2]. Pediatric injuries are the leading cause of death and disability globally and place a considerable burden on nations with limited resources [3]. Globally, more than 80% of the 875,000 yearly deaths of children under the age of 18 years occur in low- and middle-income nations [4]. Approximately 5 million children die annually due to trauma globally [5]. Research indicates that up to one-fourth of hospital admissions and approximately 15% of deaths in children in India are due to accidental injury [6]. Injuries resulting from these traumatic events result in extended periods of impairment and impose a substantial mental and financial strain on the child, family, and society [7].

The epidemiology and frequency of trauma-related accidents in the pediatric population vary from nation to nation due to variations in socioeconomic conditions, geographical features, and population characteristics [8,9], which is likely to result in a regional variation in the incidence, pattern, manner of injury, location of damage, and the result of trauma. Considering that most trauma incidents are avoidable, particularly in the pediatric age group, identifying the pattern of trauma in each location is essential for developing health-related policies and preventive strategies [8].

Data from the National Crime Record Bureau (NCRB) indicates that 15% to 20% of trauma-related fatalities occur in minors [10,11]. Children are not young adults on account of anatomical and physiological differences. The skeleton in infants is by and large cartilaginous. Softer bones in children respond differently to bending forces than adult bones resulting in fracture types that are different from adults [12]. In contrast to adults, the bones in children have a physis. An injury to the physis may lead to late deformity in the bone, which may require corrective surgery in the future [12]. While a lot of work has been done in the field of epidemiology of adult musculoskeletal trauma, there is a need to document the epidemiology of pediatric musculoskeletal trauma. A careful investigation of the epidemiology of pediatric musculoskeletal trauma can provide insight into its causation and the demography of the affected children and help us devise preventive strategies to reduce the burden of pediatric musculoskeletal trauma.

The purpose of this study was to describe the epidemiology of pediatric musculoskeletal trauma in terms of patient demography, mode of injury, site where the injury was sustained, the common injury patterns, and the common injuries sustained in different age groups.

## Materials and methods

This prospective observational study was conducted on pediatric trauma patients admitted to trauma units of the Department of Orthopedics and Department of Pediatric Orthopedics of King George's Medical University (KGMU), Lucknow. The trauma center of KGMU is a level 1 trauma center. The study included musculoskeletal trauma patients up to the age of 18 years, provided they had given written informed consent. We excluded patients for whom the medical history was likely to be doubtful. This encompassed children with suspected battered baby syndrome, those with global developmental delays, and cases of attempted suicide. We also excluded children who could not be stabilized (expired) in the emergency department of the trauma center. Written informed consent was obtained from the parents of the enrolled children up to the age of seven years. In children between 7 and 11 years of age, verbal assent was obtained from children in addition to written informed consent from the parents. In children between 12 and 18 years of age, written assent was obtained from children in addition to written informed consent from the parents. A total of 201 pediatric trauma patients up to the age of 18 years were included in the study. The study received approval from the institutional ethics committee under letter number 1073/Ethics/2022, dated August 31, 2022.

Data collection was conducted through a standardized method by the primary author, who holds an MS in Orthopedics. The data were entered on a password-protected computer. To maintain confidentiality, data regarding patient identifiers were not recorded. The data were collected once the patients had been stabilized.

We collected information about age in completed years, sex, time since the injury to presentation to the trauma center, mode of injury, the site where the injury was sustained, and the precise injury sustained. The enrolled patients were further subclassified into four age groups: 0 to 3 years, >3 to 6 years, >6 to 12 years, and 12 to 18 years. In the case of RTAs, we also recorded the exact mechanism of injury (hit, slip, or any other) and whether the accident happened on a paved road or an unpaved road. Information about hits was recorded in the following groups: pedestrian hit, cycle hit, motorized three-wheeler hit/light motor vehicle (LMV) hit, and heavy motor vehicle (HMV) hit. Slips were further subclassified as motorized two-wheeler slip, cycle slip, or any other. Information about the use of helmets was also recorded in cases of injuries sustained while riding a motorized three-wheeler. Falls were categorized as either falls at ground level or falls from a height. A subgroup analysis of the mode of injury was done using the age group and sex variables. 

Statistical analysis

The data collected were tabulated using Microsoft Excel software and analyzed using IBM SPSS Statistics for Windows, Version 21.0 (IBM Corp., Armonk, NY, USA). Continuous data were described using means and standard deviation (SD). Categorical data were summarized using frequency distribution tables and the chi-square test. An unpaired t-test was used to compare the means of continuous variables.

## Results

A total of 201 patients were enrolled in the study. Of the 201 enrolled patients, 146 (72.63%) were males and 55 (21.37%) were females. The number of males was higher in all the age groups except for in the 0 to 3 years age group. The largest number of patients (*n *= 111, 55.22%) were enrolled in the 12 to 18 years age group (Table 1).

**Table 1:** Frequency distribution of the age of the enrolled patients.

Age distribution (years)	n	%		Gender			Male-to-female ratio
				Males		Females	
0 to 3	6	2.84		2		4	1:2
>3 to 6	24	11.94		15		9	5:3
>6 to 12	60	29.85		37		23	1.61
>12 to 18	111	55.22		92		19	4.84
Grand total	201	100.00					

Of the 201 patients enrolled in our study, 112 (55.72%) were injured on the road. 67 (33.33%) at home, 19 (9.45%) at a farm, 1 (0.5%) at the workplace, and 2 (1%) at a railway crossing. Of the 201 patients enrolled in our study, 33 (16.41%) presented directly to the trauma center, while 168 patients (83.58%) were admitted to the trauma center after being referred from some other hospital. Of the 168 referred patients, 109 (54.23%) were referred from a secondary care hospital, 58 (28.86%) from a primary care hospital, and 1 (0.50%) from a tertiary care hospital.

The mean time since the injury to the reception in the KGMU trauma center was 19.13 ± 33.86 hours. Forty-seven (23.38%) patients arrived within one to six hours of sustaining an injury (Table 2).

**Table 2:** Time since injury to hospital admission.

Time since injury (hours)	n	%
<1	18	8.96
1 to <6	47	23.38
6 to <12	33	16.42
12 to <18	37	18.41
18 to 24	10	4.98
24 to <48	11	5.47
48 and more	45	22.39
Grand total	201	100.00

Of the 201 enrolled patients, 111 (55.22%) were found to have sustained an injury due to an RTA, 59 (29.35%) due to falls from height, 18 (8.96%) due to falls at ground level, 1 (0.5%) due to an assault, and 12 (5.97%) due to other causes.

There was a significant difference in the mode of injury in the different age groups. We found falls to be the most common cause of injury in children in the 0 to 3 and >3 to 6 years age groups. Falls from heights were more prevalent across all age groups compared to falls at ground level. RTAs were the most common cause of injury in children in the >6 to 12 and >12 to 18 years age groups (Table 3). Of the 111 patients who were injured due to RTAs, 87 (78.18%) met with an accident on a paved road, while 24 (21.72%) on an unpaved road.

**Table 3:** The subgroup analysis of the mode of injury on the basis of age. RTA, road traffic accident

Mode of injury	Age distribution (years)								*P*-value
	0 to 3		>3 to 6		>7 to 12		>12 to 18		
	n	%	n	%	n	%	n	%	
RTA	1	16.67	8	33.33	33	55.00	69	62.16	0.0297
Falls at ground level	2	33.33	4	16.67	5	8.33	7	6.31	
Falls from height	3	50.00	9	37.50	20	33.33	27	24.32	
Assault	0	0.00	1	4.17	0	0.00	0	0.00	
Others (Machine/Blast)	0	0.00	2	8.33	2	3.33	8	7.21	
Grand total	6	100.00	24	100.00	60	100.00	111	100.00	

There was significant difference in the mode of injury among males and females (*P *= 0.0034). We found RTAs being the more common mode of trauma in males and falls being the more common mode of trauma in females (Table 4).

**Table 4:** The mode of injury subgroup analysis on the basis of sex. RTA, road traffic accident

Mode of injury	Gender			
	Female		Male	
	n	%	n	%
RTA	19	34.55	92	63.01
Low-energy fall	7	12.73	11	7.53
High-energy fall	26	47.27	33	22.60
Assault	0	0.00	1	0.68
Others (Machine/Blast)	3	5.45	9	6.16
Grand total	55	100.00	146	100.00

Of the 111 cases of RTA, 96 (86.48%) were due to some kind of hit (pedestrians, cyclists, or vehicles being hit), 13 (11.71%) due to the vehicle slipping on the road, and 2 (1.8%) due to any other reason (Table 5). Of the 57 cases involving a motorized two-wheeler (all being over 50 cc), 29 (50.87%) were being driven by the patient. At the time of accident, of these 29, 11 (37.93%) were wearing a helmet, while none of those riding pillion were wearing a helmet.

**Table 5:** The mechanism of injury in cases where the mode of injury was RTA. RTA, road traffic accident

RTA		n	%
Hit	Pedestrian	18	16.22
	Cycle	16	14.41
	Motorized two-wheeler	49	44.14
	Motorized three-wheeler	5	4.50
	Light motor vehicle	3	2.70
	Heavy motor vehicle	1	0.90
	Any other	4	3.60
Slip	Motorized two-wheeler	8	7.21
	Cycle	5	4.50
Any other	Overturning of three-wheelers	2	1.80
	Grand total	111	100

**Figure 1:**
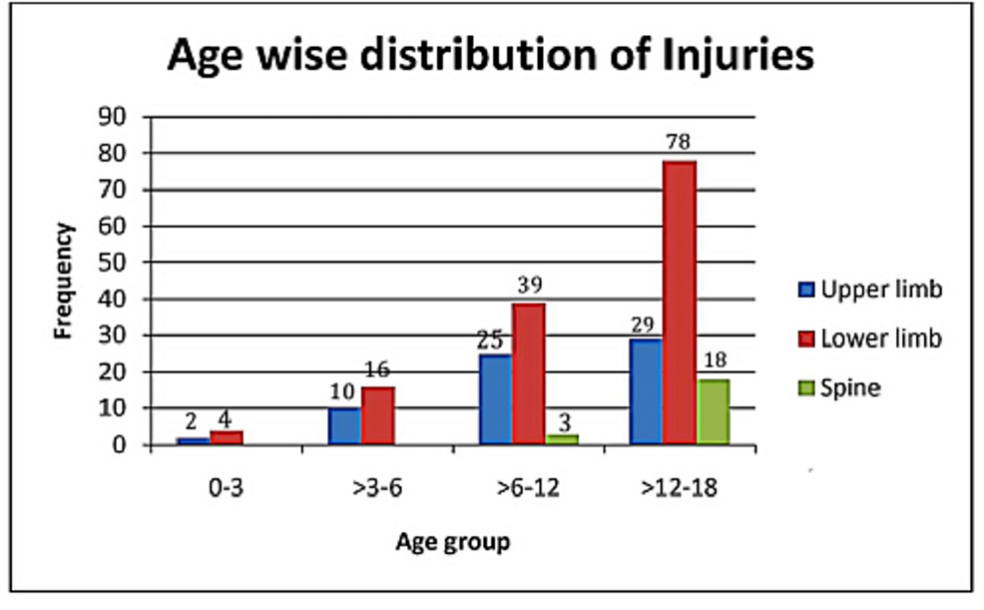
Age-wise distribution of injuries.

A total of 224 injuries were found in the 201 enrolled patients. Injuries to the lower limb were the most common in all the age groups (*n *= 137, 61.16%). No patients aged below six years experienced a traumatic spine injury (Figure 1). The share of traumatic spine injuries increased with age.

Fracture shaft femur was the most prevalent injury across all age groups. In children below 12 years of age, a supracondylar fracture of the humerus was the second most common injury. In children above 12 years of age, the second most common injury was a traumatic fracture of the spine (Table 6).

**Table 6:** Age-group-wise frequency distribution of injuries. ^*^n is the number of three most common injuries sustained in the respective age groups.

Age group (years)	Number of injuries	Three most common injuries	n^*^ (%)
0 to 3	6	Fracture shaft femur	4 (66.67)
		Fracture supracondylar humerus	2 (33.33)
>3 to 6	26	Fracture shaft femur	9 (34.61)
		Fracture supracondylar humerus	5 (19.23)
		Fracture both bone leg	5 (19.23)
>6 to 12	67	Fracture shaft femur	22 (32.83)
		Fracture supracondylar humerus	10 (14.92)
		Fracture of both leg bones	10 (14.92)
12 to 18	125	Fracture shaft femur	41 (32.8)
		Traumatic spine	18 (14.4)
		Fracture of both leg bones	16 (12.8)
Total	224		

## Discussion

This study has reported a male preponderance among the enrolled patients, with RTA as the most common mode of injury. It highlights significant differences in the mechanism of injury in males and females and in different age groups, drawing attention to instances of underage driving and the inadequate utilization of helmets among individuals injured while riding motorized two-wheelers.

In this study, male preponderance was observed among enrolled patients in all the age groups except 0 to 3 years. A male preponderance irrespective of the age group involved has been reported by other researchers as well [11-13]. A lower number of males in the 0 to 3 years age group in our study could be an aberration as the number enrolled in this age group was only six. In Indian society, male children enjoy greater freedom of movement and more opportunities to be out of home, which, in turn, increases their chances of being exposed to potential risk factors for trauma like playing on the roads. This could be the reason for the male preponderance reported by us as well as several other studies [11-12].

Several studies have reported falls to be the most common mode of injury [13-14] in pediatric trauma patients. In contrast, this study found RTAs to be the most common mode of injury, which is similar to the finding of a study by Kundal et al. [11]. The reason for this difference lies in the age at which the children were enrolled in the studies. Studies reporting falls to be the commonest mode of injury enrolled children up to 12 [13] or 15 years [14], while we had enrolled children up to the age of 18 years. An important finding of our study is that the mode of injury varied significantly with the age group in question. In the current study, RTA was the most common cause of injury in the 7 to 12 years age group (55%) and 13 to 18 years age group (62.16%). In contrast, falls from height were the most common cause of injury in the 0 to 6 years age group. Other studies have also reported that the causality of injury varies with the age group involved, with the proportion of RTAs as the prevailing mode of injury progressively rising with age [12-13]. 

An important finding of our study is that there is a significant difference in the mode of injury among males and females. We found RTAs to be the most common mode of injury in males and falls to be the most common mode of injury in females. Kundal et al. [11] have also reported RTAs and falls to be the commonest cause of injury in male and female pediatric trauma patients, respectively. As the females in our culture tend to stay at home [15], they are less likely to be exposed to RTAs. This could be the possible explanation for falls being the most common mode of injury in females.

Most of the children in this study sustained the injury at the roadside. This is in contrast to several studies that have reported the home to be the most common site where children get injured [13,14]. The difference again can be attributed to the age at which the children were enrolled. We included children up to 18 years of age, while others included children aged up to 12 years [13,16]. As age increases, children are more prone to being outdoors, thereby increasing the risk of sustaining injuries from road-related incidents. A study conducted in India has reported that the majority of children in RTA cases were riding pillion on a motorized two-wheeler when the injury happened [16]. This is expected as the study enrolled children up to the age of 12 years, who are likely to ride pillion as the legal age for driving in India is 18 years.

It is said that the outcome of a trauma patient is largely dependent on the care provided during the initial one hour. This basic concept is kept in mind while designing trauma care systems all over the world. This study has reported that none of the patients were admitted to the trauma center within one hour of sustaining the injury. The delay in admission to the trauma center might be attributed to various factors, including limited parental awareness, lack of trust in government hospitals, an ineffective prehospital transport system, or the considerable distance to the hospital. We are unable to provide a reason for this delay, as it falls outside the scope of our study. We, therefore, recommend a study that can identify the factors that govern the duration between injury occurrence and arrival at the trauma center.

This study has reported a collision or a slip of motorized two-wheelers as the most common cause of RTAs. An important finding of this study is that about one-third of all cases of RTAs were due to pedestrian or cyclists being hit by speeding vehicles. Literature reports that a large amount of injury burden due to RTAs in developing countries is incurred by the pedestrians, cyclists, and motorcyclists [17-19] as they constitute the majority of the road users in developing countries. In contrast to LMVs and HMVs, these groups are exposed to the vagaries of the traffic environment. Implementation of protective measures could result in a substantial decrease in admissions, subsequently reducing both mortality and morbidity rates.

In India, the legal age of driving is 18 years (except for 50 cc bikes for which the age is 16 years). An alarming finding of our study is that 29 (50.88%) patients sustained an injury while driving a motorized two-wheeler (all of which were above 50 cc) despite the legally mandated driving age in India being 18 years. There is no scientific data on the incidence of underage driving in India but newspaper reports indicate a high prevalence [20]. In countries where teenagers are permitted to drive, it is well-documented that teenagers exhibit higher crash rates compared to adults [21]. We propose rigorous enforcement of regulations to deter underage driving, considering its illegality; such actions could contribute to a reduction in the frequency of accidents.

It is mandatory for motorized two-wheeler riders to wear helmet in India. An alarming finding of our study is that of the 57 patients who sustained an injury while riding a motorized two-wheeler, only 11 (19.29%) were wearing a helmet at the time they sustained the injury. Self-reported use of helmet is known to be higher than actual use [22]. In our study, the information about use of helmets was collected from the patients or the attendants, and therefore, the true percentage of those using a helmet may be even lower than the 19.29% reported by us. We recommend a strict implementation of helmet rules as the use of helmets is known to result in lower mortality [23]. 

A limitation of our study is that it is hospital-based study conducted in the trauma center of a tertiary care center. Being a tertiary care center, a very large percentage of the admitted patients (83.58%) were actually referred from other centers. Therefore, the results of our study may not hold valid for primary and secondary care hospitals catering to pediatric musculoskeletal trauma patients. Another limitation of our study is that we excluded patients with *doubtful history*,* *and therefore, this study is not representative of non-accidental injuries.

## Conclusions

This study presents baseline epidemiological data on pediatric musculoskeletal injuries distributed by age groups, gender, mode of injury, site of injury, and region-wise distribution of injuries. The data may be used by policymakers in planning a pediatric trauma care system in India. The results of our study may be used to design studies to generate epidemiological data on pediatric injuries in greater detail. 
